# A qualitative insight into informal childcare and childhood obesity in children aged 0–5 years in the UK

**DOI:** 10.1186/s12889-018-6131-0

**Published:** 2018-11-06

**Authors:** Eleanor Diana Lidgate, Bai Li, Antje Lindenmeyer

**Affiliations:** 1 College of Medical and Dental Sciences, University of Birmingham, Birmingham, West Midlands UK; 2 Institute of Applied Health Research, College of Medical and Dental Sciences, University of Birmingham, Birmingham, West Midlands B15 2TT UK

**Keywords:** Childhood obesity, Childcare, Qualitative study, Health behaviour, Informal care, Grandparents, Pre-school children

## Abstract

**Background:**

Previous studies in various countries have found that informal childcare (provided by relatives, friends etc.) was associated with an increased risk of obesity in children aged 0–5 years. However, no qualitative research has been done to explore possible reasons for such a relationship and potential interventions to tackle it. We conducted a qualitative study with both parents and informal carers to explore their 1) experiences in receiving or giving informal childcare for British children aged 0–5 years; 2) perceived explanations of the relationship between informal childcare and childhood obesity and 3) preferred intervention ideas and delivery strategies for preventing obesity among those children under informal care.

**Methods:**

Four in-depth focus groups with a total of 14 participants (7 parents, 7 informal caregivers) were conducted in Birmingham and Edinburgh (1 parent group and 1 informal caregiver group in each city). Data were audio recorded, transcribed verbatim and analysed using a thematic approach.

**Results:**

The significance of informal care to parents, carers, and society was recognised (theme one). Informal carers were identified to have practical and emotional support roles for the parents (theme two). Informal care was perceived to contribute to childhood obesity in four ways (theme three): cross-generation conflict preventing adoption of healthy practices; the trade-off for parents between receiving childcare and maintaining control; reduced energy capacity of carers; and increased snacking. Potential intervention ideas and delivery strategies (theme four) were identified. Examples of identified ideas included providing carers with up-to-date weaning advice, and suggestions of healthy snacks and ways to increase physical activity level in informal care. The suggestion of utilising existing primary care platforms (e.g. health visitor check-ups) to reach and deliver low-cost information based interventions, to all children aged 0–5 years who receive informal care, was highlighted.

**Conclusions:**

This exploratory qualitative study provided novel insights into potential explanations for the evidenced link between informal care and childhood obesity in children aged 0–5 years, despite a small size and limited participants in each focus group. Our findings support the idea of and inform the development towards an information based and low-cost intervention delivered through existing primary care platforms.

## Background

Childhood obesity has become a global epidemic. In the UK, year on year the number of children who are either overweight or obese is increasing, and the age at which the onset of obesity occurs is reducing [1]. Approximately one in four children are overweight or obese by the age of 3 years [2]. There is an urgent need for more research and policies targeting the prevention of obesity in children aged 0–5 years [3, 4]. The early years are an important time for the development of healthy habits. These include an active lifestyle, a low intake of unhealthy snacks and sufficient sleep time – all of which are protective factors against obesity [5–7], and can be influenced by carers in the early years [8]. Since the majority of young children receive some form of childcare [9], this period is a crucial target area for obesity prevention, and for the creation of healthy habits [10].

Informal care - care provided by grandparents, friends, neighbours, nannies [2, 11, 12] - is a popular childcare choice in the UK. By 2010, at least a quarter of British children under the age of three were in informal care, and around three-quarters of informal caregivers were grandparents [2]. There is an increasing body of evidence, including a range of study designs from a number of different countries, to suggest that children in informal childcare are more likely to be overweight or obese than their counterparts in parental care [2, 11, 13–19]. A recent systematic review of the relationship between childcare settings and risk of overweight and obesity, found that before the age of 3 years, informal care was associated with significantly increased BMI or likelihood of overweight and obesity when compared to parental care [19]. However, no qualitative research has been done to explore possible reasons for, or potential interventions to tackle, such a relationship. In addition, according to published reviews of interventions aimed to prevent obesity in young children [3, 20–25], there are currently no interventions specifically designed to encompass informal caregivers of children under five [26]. Previous interventions targeting the early years were often delivered to children or their parents through formal platforms such as child care centres [20]. Further investigation into the obesogenic aspects of informal care is necessary and is at the centre of informing future childhood obesity preventative measures [19]. The success of these measures relies on in-depth understanding of the needs of informal caregivers [8] and parents who use informal care.

Therefore, in order to inform future development of tailored interventions to prevent obesity in children under five in informal care, we conducted a novel qualitative study with parents and informal caregivers. The aims of this study were to explore their 1) experiences in receiving or providing informal childcare; 2) perceived reasons behind the relationship between informal childcare and childhood obesity and 3) favoured intervention ideas and delivery strategies for preventing obesity in those children.

## Methods

### Setting

Informal childcare givers and parents of children under five who were (or used to be) in informal care were recruited into focus groups between September 2016 and January 2017. Participants were recruited from two major cities of the UK (Birmingham and Edinburgh) to provide a range of socio-demographic perspectives.

### Participant selection and recruitment

Participants were recruited using a purposive sampling method. Initial recruitment strategies included advertising via a grandparent carer charity, a childcare website and a mailing list of staff at the University. Inclusion criteria included: (a) informal carers or parents who were providing/using or had used/provided informal childcare, (b) the child was aged 0–5 years at time of care, and (c) willingness to participate in a focus group. There was no time limit on how long ago the care took place. All participants had a comprehensive information sheet emailed to them prior to attending a focus group, and had a chance to ask questions. Interested and eligible participants were contacted to arrange a convenient time and place to run the focus group. For practical reasons, participants read and signed an informed consent form at the beginning of the focus group. Each participant received a £10 voucher as a token of appreciation for taking part, and parking expenses were compensated.

Slow and difficult recruitment was anticipated, and occurred, owing to the unique characteristics of the research population (i.e. informal carers had no or minimal connections with any formal childcare organisations or settings). A number of strategies and media were applied to overcome the challenges in participant recruitment. These included: advertising via local baby and toddler groups; and putting up posters in community centres.

### Data collection process

Focus groups were chosen as the data collection method as they encourage interaction between participants [27]. This interaction allows the researcher to elicit people’s understandings and views, or to explore how these are advanced in a social context [28]. Focus groups were held in locations chosen by the participants, two occurred in University meeting rooms and two in participants’ own homes. Parents and carers were invited to separate focus groups to encourage open discussions of shared experiences. A semi-structured topic guide for the focus groups was created in line with the project aim and objectives. This was piloted in a practice focus group with five participants to test and refine the design of our topic guide and questions. The main questions are summarised in Table 1. The wording and the order of the questions were adapted flexibly at each group depending on the natural flow of the discussions and the identity of the group. A single researcher moderated both the pilot focus group and the four focus groups involved in this study. One of the focus groups was co-moderated with another researcher. Focus groups began with friendly ice-breaking questions to help all participants feel comfortable before moving on to more specific and targeted questions. Member checking occurred at the end of each group to ensure that the participants agreed with the moderators’ interpretation of the main concepts discussed. Focus groups lasted between one and 2 hours. All discussions were audio recorded with consent on a password-protected device. The moderator made field notes after each focus group, to record the key concepts that arose and participant interaction, and to aid transcript coding.

**Table 1:** Summary of Focus Group Topic Guide

1. What do you think informal care is? What types of informal care have you used or given and why?	
2. Carers: Can you describe your typical day with the child you look after? Parents: Can you describe a typical day your child will have with the carer? (Making special attention to Who? What? Where? When? Why?)	
3. Do you receive/give any suggestions as to how the child should be looked after in relation to health or health behaviour?	
4. Do you think that there is a problem with pre-school aged children being overweight?	
5. What knowledge or support do informal caregivers need to help prevent obesity in the children they care for?	
6. Moderator provides existing evidence on the relationship between informal childcare and obesity. Can you think of any possible explanations for this evidenced association?	
7. What are parents and informal caregivers perceptions of potentially feasible and effective intervention targets and ideas^a^? (Making special attention to Who? What? Where? When/how often/how long? )	
8. What would be feasible ways to recruit suitable participants for future questionnaire surveys that aim to research this topic further?	
9. Summary and closing	

^a^Focus group participants were not provided with any examples of potential interventions, in order to generate an open discussion with fresh ideas that were not influenced by the moderators’ knowledge in the field.

Data collection was continued until a range of responses had been collected in the different focus groups; however it was not deemed necessary to achieve data saturation as our analysis aimed to capture parents’ and carers’ experiences and understandings rather than develop theory [29].

### Data analysis

Given a scarce knowledge basis on why informal care is linked to childhood obesity, an inductive thematic analysis approach was chosen to analyse the data set, as described by Braun and Clarke [30]. Focus group recordings were anonymised and transcribed verbatim. Each participant was assigned a unique ID relating to their identity and group number (for example PG1M1: Parent Group 1 Mother 1; CG1G2: Carer Group 1 Grandparent 2); this ID is reported alongside the relevant quotes in the results. To aid the generation of initial codes, the first author became familiar with the data by repeatedly reading the transcripts. Initial codes were produced systematically with the help of NVivo computer software [31]. A coding book was first developed by the first author, with guidance and contributions from the other authors, based on the richest dataset. This was expanded and refined as it was applied to the rest of the transcripts. The authors made use of visual representations, such as mind-maps and theme piles (codes on small pieces of paper which are arranged with similar codes), to develop initial higher-level themes from the descriptive codes and to begin to identify those with added significance. To increase the authenticity and credibility of the results, analyst triangulation, with three researchers from a range of backgrounds, was adopted in the final stages. This involved reviewing the finalised codebook and reaching a group consensus on the definition and content of the final themes. The analysts ensured that the dataset within each theme was connected whilst also being sufficiently different to the data within the other themes [32]. In order to minimise researcher bias, a reflexivity journal was maintained by the author to reveal their thinking process behind the development of codes and emerging patterns, and to reflect on how their opinions may impact on the analysis.

## Results

A total of 14 participants took part in four in-depth focus groups, including seven parents and seven informal care providers (Table 2). Two focus groups were run in Edinburgh, one group with four mothers, and the other with three informal care providers. The remaining two groups were run in Birmingham, one consisting of three mothers and the other with four informal care providers.

**Table 2:** Participant demographics

	Birmingham		Edinburgh	
	Parent (*n* = 3)	Carer (*n* = 4)	Parent (*n* = 4)	Carer (*n* = 3)
Age				
60 or older	0	2	0	1
50–59	0	1	0	2
40–49	0	0	0	0
30–39	3	0	4	0
20–29	0	1	0	0
Gender				
Female	3	3	4	3
Male	0	1	0	0
Ethnicity background				
White	3	3	4	3
Black	0	0	0	0
Asian	0	1	0	0
Employment				
Full time	0	1	0	0
Part time	3	1	4	2
Unemployed	0	0	0	0
Retired	0	2	0	1
Annual household income				
No income	0	0	0	0
< 30 K	0	2	0	0
30-60 K	4	1		1
60-100 K	2	0	1	0
> 100 K	0	0	0	0
Did not specify	0	1	0	1
Level of education				
University/College	3	4	4	3
Secondary school	0	0	0	0
Primary school	0	0	0	0
Relationship of carer to child				
Parent	3	0	4	0
Grandparent	0	3	0	2
Nanny	0	1	0	0
Child-minder	0	0	0	1
Age of the child (mean in years, range)	2.70 (0.75–5.00)	3.90 (2.00–5.00)	2.90 (2.00–4.00)	2.80 (2.25–4.50)
Length of time the child was in informal care (mean in years, range)	1.20 (0.25–2.25)	3.20 (0.50–5.00)	2 (0.50–4.00)	2.40 (1.25–4.00)
Number of days a week the child was in informal care (mean in days, range)	1.00 (1.00)	3.80 (1.00–7.00)	1.20 (0.25–2.00)	3.00 (2.00–5.00)
Average number of hours a day the child was in informal care (mean in hours, range)	8.00 (6.00–10.00)	6.80 (3.00–10.00)	6.30 (4.00–9.00)	6.90 (5.00–9.50)

Four core themes emerged from the analysis: (1) the importance of informal care to families and society; (2) practical and emotional roles of informal carers; (3) potential explanations for the link between childhood obesity and informal care and (4) potential intervention opportunities and strategies. Detailed results are presented below and illustrated with quotes.

### Theme 1: The importance of informal care to families and society

Both parents and carers consistently discussed how vital informal care was to both their family and to society.

All parents reported that the main reason they used informal care was to allow them to work, and that informal care had the advantage of being flexible. In most cases, informal care was unpaid. This was an important consideration for parents when deciding what type of child care to use. Formal care was generally thought to be very expensive and often parents had no choice financially but to go back to work, and relied on informal carers:

*“It was sort of to help with the cost if you’ve got 2 children we didn’t put them both into nursery for 3 days a week so my mum ended up having both children when I came back to work”* (PG2M1).

Informal care usually stems from an existing relationship, e.g. with a family member or friend, which means that parents know and trust the carer. All the parents noted this as another reason for choosing informal care over formal care:

*“Certainly I wouldn’t be happy passing him to somebody I didn’t know and in a nursery that was one of my main concerns cos the staff turnover can be quite high”* (PG1M3).

Informal care also means a lot to the carers themselves. For the nanny and the child-minder in this study, the childcare they provide is their form of employment. Whereas with grandparent carers, both parents and grandparents stated that the care arrangement was a whole family decision and that the grandparents offered to care as they wanted to help:

*“I didn’t mind cos I prefer to look after her and know that she’s being looked after properly and being treated properly and happy”* (CG1G1).

*“Oh no I wouldn’t miss it [caring for her grandchild] for the world”* (CG1G2).

In addition to being important to both the parents and the carers, one participant gave insight into how necessary informal care is to society:

*“Grandparents care of children… is worth about fourteen point seven billion to the state every year because people are able to work and all that type of thing, we are a really useful resource”* (CG1G3).

### Theme 2: Practical and emotional roles of informal carers

All participants recognised and agreed that a large part of the informal carers’ role is to provide practical support to both the parent and the child. Carers noted that it is an ever-changing role and can be either rigid or fluid in structure, depending on the carer. Some of the key responsibilities of the carers are:

*“I get the children dressed”* (CG2G4).

*“They’ve got gymnastics class booked so granny takes (name of daughter) to that”* (PG1M1).

*“No mum’s gonna go for me because ((laughs)) so my informal child carer is going to go to his 27 month check, so that’ll be quite good”* (PG1M4).

Not only do carers have a role in practical support they also play a part in emotionally supporting the parents. One mother, of twin’s aged 2 and a 4 year old, used grandparent care to take her children to classes and clubs to give her a break for a couple of hours on the days she was not working. Informal care relieves pressure on parents in two ways: the first being that the parents know their children will be safe:

*“They were well looked after and I didn’t have to worry”* (CG1G1).

The second is to allow parents to work to relieve financial pressure:

*“It’s to enable them to continue their careers when they really needed to work just to pay their mortgage”* (CG2G1).

In addition parents look to their parents for advice and support with parenting as they have done it before. With increasing numbers of people living away from their families, emotional support, via the telephone, is often the only support grandparents can give if they live far away:

*“With my mum even though she wasn’t there all the time she’d phone me and I was upset ‘oh is she still not sleeping just give her some baby rice’”* (PG2M2).

### Theme 3: Potential explanations for the link between childhood obesity and informal care

Participants were asked to comment on reasons why children in informal care might be more likely to be overweight or obese. Four potential explanations provided by participants are presented below.

i) Cross-generation conflict preventing adoption of healthy feeding practice in family

This was a common experience and predominant topic discussed by all mothers in one of the parent focus groups. It refers to the battle parents had between current recommendations, and the previous experience and opinions of older caregivers in the family:

*“I would definitely tell my mum how I’d want them to be fed especially when they were younger only because we had different views about weaning and stuff like that”* (PG2M1).

*“Cos we did baby led weaning that was a bit controversial with both grandmas so particularly my mum there was a lot of ‘well shall I just give him some puree that I bought from the shop’ and (the participant replied) ‘I’d rather you didn’t’ sort of thing”* (PG2M3).

As mentioned in Theme two, parents looked to their parents for advice and support, especially regarding breastfeeding and when to introduce solid foods. Unfortunately grandparents often had out-dated opinions regarding these topics (e.g. embracing bottle feeding and encouraging early weaning) as medical advice had changed over time, but they wanted to influence parent choices:

*“My mum actually encouraged me to wean early which I think a lot of mums do because (name of daughter) was struggling and she wasn’t sleeping… so I did start a little bit early”* (PG2M2).

In some cases the conflicting beliefs between generations in childcare practice put enormous pressure on parents to adopt undesirable feeding behaviour:

*“My mum was like ‘well give him some baby rice give him some baby rice’ and I remember at 5 months I was pushed that hard that I offered him baby rice and he didn’t want it and I was like ‘see he doesn’t want it’”* (PG2M1).

When parents were adopting a health guideline recommended practice, such as breastfeeding, that the grandparents did not believe in, or did not do with their children, it was hard for grandparents to provide support. This made the adoption of healthy feeding behaviours among the parents very difficult:

*“No my mother-in-law was the same yeh she didn’t agree with breastfeeding at all… it was really hard cos she was the only other person I had as support apart from my husband”* (PG2M2).

There was a feeling that grandparents might also take this change in practice as a personal attack:

*“I think with her it’s because she bottle fed me and my sister and I think she felt it was a bit of a personal well are you saying it wasn’t good enough what I did”* (PG2M3).

The constant pressure that was put on some parents by grandparents meant that they had to constantly justify their decisions why they had chosen to do something differently:

*“And it’s really hard cos you don’t trust your own instincts do you… I used to have to have a book to sort of back it up… because otherwise you do feel so peer-pressured that you have to justify yourself don’t you as to why you do things”* (PG2M1).

Despite all of the above, the parents reported that they had the final say as the carers respected their decisions. However, they described that this respect did not come easy and the parents had to be strong-minded and stubborn to get what they wanted. Interestingly, this respect for parents may be cultural. British grandparents were reported to respect the parent to have the final decision, whereas grandparents from other countries may not:

*“Do you know what that’s quite interesting cos my mum’s not English, no my mums Dutch and they are very forthright people and she is less respectful of my opinions than my mother-in-law who is English”* (PG2M3).

Although grandparents might promote bottle-feeding and early introduction of solid foods, their intention might be to be more helpful for the parents. For example bottle-feeding would allow other family members to feed the baby and to give the mother a break:

*“I don’t think she (referring to her mother) does believe that bottle feeding is much better but that it is a lot easier, it means she could have helped so like especially when I used to have it really rough in the nights” (PG2M5).*

ii) Trade-off between receiving childcare support and maintaining control

As mentioned in Theme 1, an informal care arrangement usually arose from a relationship. This theme refers to the balancing act parents faced between maintaining that relationship yet also promoting healthy weight in their child. In terms of the trade off, parents received care for their children, but in exchange lost control over what the child ate as they felt unable to give healthier suggestions, as they did not want to affect their relationship with the carer. This was a common theme that arose in all parent groups but not discussed in the carer groups, suggesting that caregivers may be unaware the parents felt this way:

*“Yeh rock the boat or be too critical, it’s the relationship that is there as well as the kind of helping you yeh giving you care so it would be very difficult”* (PG1M4).

In some cases the parents relied on the care so much that they wanted to make it as easy as possible for the carer, so they did not feel comfortable giving suggestions regarding their feeding or activity. Parents also felt indebted to their informal carer and did not feel they could criticise what they were doing:

*“You know my mum is doing me a huge favour by helping me out with this I can’t really say to her ‘come on down and I’ve got something stuck to the wall”* (PG1M3, referring to an intervention suggestion given by another parent in the focus group).

Parents were also wary that suggestions might be taken as an insult and it might look like the parent did not trust the carer to feed their child. One mother also stated that she would feel more comfortable disagreeing with someone she did not know, as there was no relationship to think about:

*“Yeh find that easier to say to someone I’m paying for it, but when it is a favour yeh to help you out its harder to say what you really want maybe”* (PG1M1).

As one mother-in-law used to work in a nursery, she was likely to have had experiences of parents providing feeding suggestions for their children, so she was more receptive to suggestions:

*“I know that she wouldn’t leave him crying because she knows that I don’t and it’s not her child and she is very good like that so maybe that does actually come from working with other people’s children and being used to that advice”* (PG2M3).

iii) Fewer opportunities for physical activity

This was a common perception across all stakeholder groups (i.e. both parents and carers). Childcare is a demanding task. Parents were younger and had more energy to keep up with children and keep them engaged in more active play. In contrast, most informal carers were grandparents who were older with a natural decline in energy levels. In addition, some grandparent carers in this study were still working; this could also lead to exhaustion:

*“Certainly if grandparents as they are shortening their working day but they’re still at work to look after the kids then I think they’re probably knackered”* (CG1G3).

As a result, screens, such as televisions and tablet computers, were often used as a way of entertaining or distracting children in order for the informal carer to get along with household tasks:

*“I think as people get older they do get tireder and looking after kids as you get older is harder work you are more likely not to play with them you are more likely to… say ‘go and watch the tv or what about your computer or even do some drawing’ rather than playing outside”* (CG1G3).

Parents recognised that their children would be more physically active if they were in formal care, and that activity levels were at their lowest in the winter months in informal care due to the weather. Equally, parents were aware that formal facilities were set up for outside play for all months of the year with toys under rain shelters and activities centred on the weather, so children were active throughout the year. In formal care:

*“You wouldn’t have those hours where they would be sat down”* (PG1M1).

iv) Increased snacking

One common view among parents was that informal care, especially grandparent care, was more lenient. One grandparent gave a possible explanation for this:

*“I think when it’s your grandchildren they say ‘please granny can I have it’ it’s very hard to not to be totally sort of ‘nope’”* (CG2G1).

Multiple participants, including both parents and carers, were aware that grandparents treated their grandchildren with sugary foods such as sweets and chocolates:

*“Well I must say I always had to prevent granddad buying packets of sweets… he would be thinking he was being kind”* (CG2G2).

### Theme 4: Potential intervention opportunities and strategies

Participants discussed potential strategies that would help parents gain support or understanding from grandparents/carers, and strategies that would support carers to promote healthy behaviours in the children in their care. Participants provided suggestions for implementation strategies related to the questions of ‘why’, ‘what’, ‘how’ and ‘when’ interventions should be delivered.

i) Why are interventions needed?

A common theme that emerged across all groups was that grandparents’ knowledge might need updating. As parenting and childcare advice change regularly, it was believed that grandparents’ knowledge was out of date owing to the fact that most of them raised their children decades ago. Specific learning needs were highlighted, including for example an update on nutritional advice (e.g. when to wean), and an understanding of the influence of time spent in front of screens. As parents and grandparents comment below:

*“People will argue ‘oh well you’ve done it already because you had your own kids’ but actually society and we’ve talked about the screens and so on has changed so much”* (CG1G3).

*“Yeh there’s always different sort of nutritional advice changing and it’s always handy to know the up to date information”* (PG1M2).

All parent groups discussed a second reason for educational intervention. Parents mentioned that when they wanted to choose a specific way for childcare/feeding, and ask grandparents to follow, it would be useful to have ‘back-up’, whether it was from professionals or in the form of a leaflet. Having this support from an outside source was repeatedly stated by parents as a positive way of decreasing the pressure from grandparents and as a gentle way of encouraging healthier habits. Parents felt that if grandparents were equipped with the knowledge then they could better support the parent in the decision they made.

*“It’s almost like back up you say ‘this is the way I want you to do it, this (imitating a leaflet) is where I got the information from’ it might be quite helpful”* (PG2M2).

ii) What should be included in the intervention?

The participants also recognised that the intervention programme must be tailored to the different needs of the child at different ages and suggested that the content of the intervention should be divided into information for: 1) zero to 5 years; 2) under 1 year; and 3) over 1 year.

Information that was considered useful for all children aged 0–5 years included: recipe ideas, cookery lessons, activities to keep children entertained while carers are trying to do tasks, and signposting of events and days out in local area:

*“New recipes… to help people think of different things to do for their children”* (CG2G1).

*“Say random days out and things to do”* (PG1M1).

In terms of information for children aged one and under, participants suggested including: weaning advice, healthy snack suggestions, and a reference guide about sugar content in common drinks. It was preferred that advice at this age was mainly focussed on healthy eating and feeding:

*“Healthy snacks is always a good thing, I think carers, not in my case, they’re the ones that ‘oh well give him a biscuit give him a bag of crisps’ you know cos you just run out of ideas sometimes”* (PG2M2).

*“And like about the drinks and stuff and… sugar in drinks”* (PG2M1).

Participants suggested including the following information for children aged one and over: ways to increase activity level in informal care, and directing children away from screen time:

*“Once they get to sort of from 3 to 5 I think you really need to focus on steering away from like tablets which are an easy thing to do”* (PG2M1).

iii) How can the intervention be delivered?

With regard to the question of ‘how’ interventions should be delivered, all participants welcomed the format of either workshops or leaflets for carers, containing the information listed above. However, both stakeholder groups recognised that a potential challenge with workshops is that people would not attend. It was also agreed that physical resources were better than Internet ones and that materials should be addressed to and target carers but re-phrase the word ‘informal’ as it may imply that the carers are not good enough. It was also suggested that communication should not make anyone feel targeted or criticised, and participation in any workshops should be voluntary and include the children as well:

*“Even like a little leaflet book and it’s actually for carers outside parents so that you could give it to them and they could flick through it in their own time”* (PG2M1).

*“Make it friendly and not as if they’re being criticised”* (CG2C1).

All parents shared the opinion that the information for carers is better provided from an outside source than from the parent themselves. They agreed that the education would be better coming from a healthcare professional, as grandparents would take the information more seriously, especially if their views were being challenged. Moreover, parents stated that they preferred to hear information and advice from healthcare professionals who were also parents themselves, as they understood better what the parent was going through. Parents stated that this would probably be the case with their informal carer too:

*“I think it kind of would make it less awkward to approach my mother if ‘oh (name of son) got this at nursery and it’s for grandparents’”* (PG1M3).

*“I think if it’s somebody like a health care professional or somebody that is sort of qualified at the children’s centre I think they’re more likely to take what they say is the right thing”* (PG2M1).

iv) When and where should the intervention be delivered?

All study participants were aware that current and prospective informal carers and parents of children aged 0–5 years are very difficult to reach because they have little or no engagement with a formal site or institution. It was agreed that future interventions targeting this population should utilise existing primary care platforms to deliver an intervention with maximal reach and minimal costs. Existing and population-wide platforms include, for example, antenatal and postnatal health visitor appointments, child development check-ups or national routine vaccination appointments:

*“The health visitor could say oh you know ‘who’s going to look after your child’… so that could be through a health visiting situation they could feed out you know because they would be talking to them hopefully… but you know if people are maybe thinking about going back to work just feed that into the information that’s available from a year on if you are going to have (an informal carer} these are the packs you can get”* (CG2C1: Carer Group 2 Child-minder 1).

The participants also suggested using radio, newspapers and social media to distribute educational and supportive information on healthy weight promotion among children under 5 years in informal care, to reach people of all socio-economic classes.

## Discussion

Informal childcare is a popular choice for British parents. Despite the well-documented link between informal childcare and childhood obesity in children aged 0–5 years, no studies have explored potential explanations for this link. Moreover, no intervention programmes have been designed specifically for children outside formal care. This UK-based exploratory qualitative study explored the informal childcare arrangement from both parents and carers’ perspectives. It obtained insights into possible explanations for the relationship between informal childcare and obesity among children aged 0–5 years. Moreover, potential intervention opportunities and delivery strategies to support informal carers and parents to promote healthy weight in those children were also identified.

The importance of informal care to families and society was highlighted. We have identified that carers want to care, and they value the special bond they develop with the child in their care. This reasoning for caring was found in a previous study [33].

Informal carers were identified to provide practical, emotional and financial support for the family. Informal childcare, especially that provided by grandparents, is appreciated by parents and is an important source of financial support that permits parents to undertake paid employment [34]. In addition, grandparents may be able to spend more time with their grandchildren than the parents, enabling good social and emotional wellbeing in the grandchildren [26].

Four potential explanations for the evidenced link between childhood obesity and informal care were identified. The first relates to cross-generation conflict preventing adoption of healthy feeding practices within the family. Our findings have shown that grandparents may be influencing parents with out-dated information due to personal experience or preference, especially regarding breastfeeding and weaning. Initial breastfeeding [35, 36] and baby-led weaning (as evidenced by case-control [37] and cohort studies [38]) have both been noted as significant protective factors against obesity in children. Formal and informal childcare is associated with a reduced likelihood of breastfeeding, when compared to parental care [2]. The influence grandparents have on breastfeeding initiation and early introduction of solid food is significant and is well documented in the literature. A recent systematic review of 13 studies from a range of low and high-income countries, found that if grandmothers had their own breastfeeding experience or were positively inclined towards it, then this would have a positive impact on the mother breastfeeding [39]. In addition, a German cohort study of 3822 mothers noted that if the maternal grandmother had a negative attitude towards breastfeeding the mother was up to 3.62 times more likely not to initiate breastfeeding [40]. Our finding that lack of emotional support from grandparents, due to differences in opinion, can have a negative impact on breastfeeding initiation and continuation is supported by the literature [41, 42]. If friends, and family perceive that the mother should exclusively breastfeed the infant, the mother’s intention will be the same as that of the people around her [43]. Another reason for early weaning has been hypothesised. Grandparents often care for a group of children of different ages, so they may be more likely to encourage early introduction of solids to make mealtimes easier [14, 16].

Participants described that despite instances of cross-generation conflict, grandparents respected the parents to have the final decision. However, participants reported this respect was cultural, with British grandparents being more respectful than those from other cultures. For example, in Chinese culture the infant feeding preferences of significant others in the family (especially the mother-in-law) are often followed by new mothers, even when they are different to the mother’s desires [44]. Recognising the impact of culture on parental and family decisions is of importance when designing future interventions, as Britain has a culturally diverse population.

A second potential explanation for obesity in informal care relates to the trade-off between parents receiving childcare for their children and losing control over feeding and activity choices. This trade-off has also been documented in two qualitative studies in the US. One of those reported that parents felt they had limited ability to control grandparents’ feeding practices as they relied on their care [45]. The other reported that mothers who accept support from their mothers may lose control over the food their children eat [46]. Future interventions should recognise this trade off that parents are balancing and should try to minimise the cross generation knowledge gap in childcare practice (as evidenced by this study) by providing the necessary knowledge and skills that grandparents need. This would help parents in receiving the valuable support from grandparents in terms of childcare without compromising the relationship.

Reduced energy capacity of informal carers, leading to decreased activity levels in the children, has been identified as a third perceived cause of childhood obesity. Parents consistently stated that their children were less active in informal care compared to formal care. This is a consistent finding with current British literature [20]. Interventions should incorporate a physical activity component, as physical inactivity and sedentary behaviours, including television watching, are repeatedly reported in the literature as being linked to childhood obesity [47–50]. Interestingly a mixed methods study, including both qualitative and cross-sectional aspects, conducted in China, offered a different explanation for decreased energy levels in informal care [51]. The study reported that due to the single-child family structure, grandparents tended to overprotect their grandchildren from household chores, therefore limiting their physical activity levels.

Increased food consumption in informal care was identified as a final perceived cause of obesity. These findings are in line with literature from Asia. A Japanese Cohort study found that compared to parental care, children who were cared for by grandparents at the age of 3 years had a higher prevalence of snacking and subsequently had a higher mean BMI over time [52]. Cross-sectional data of dietary habits and physical activity of 497 schoolchildren in China found that children who were primarily cared for by a grandparent consumed over two or more portions of unhealthy snacks per week than those children who were primarily cared for by their parents or other adults [51]. Qualitative data from the same study reported that grandparents overindulged their grandchildren and had misperceptions about what comprises a healthy diet in children.

The final theme of this study referred to potential intervention opportunities and strategies.

We identified that in order for informal carers to provide appropriate support to parents and to encourage healthy habits, their knowledge needs updating on current best practices. Grandparents have been reported to be the second most commonly cited source for information, after health visitors [53]. As the majority of informal carers are grandparents [2], this highlights the need to target up to date information and advice to this group. Participants identified that a way of achieving this could be via a brief intervention, centred on a leaflet, targeted to carers with the aim of preventing obesity in children in informal care. An intervention feasibility study that delivered an antenatal session centred on a leaflet written specifically for fathers and grandmothers about breastfeeding, found this to be acceptable, useful and enjoyable by all participants [54]. In addition, a recent English randomised control trial found a low-cost opportunistic 30-s brief intervention, delivered by primary care physicians, was acceptable to patients and an effective way to reduce weight in patients with obesity [55]. This intervention strategy could be adopted by future interventions targeting informal carers and adapted to include more topics related to healthy weight promotion in young children.

Adopting this approach would mean an existing primary care communication platform (such as a community midwife, health visitor or GP appointment) could be used, making it virtually cost neutral. Most previous interventions that aimed to prevent childhood obesity were labour intensive and required certain equipment or facilities. However, none of the recently completed large trials of childhood obesity prevention programmes in the UK showed evidence of effectiveness [56, 57]. Involving informal carers in routine antenatal and postnatal appointments provided by the NHS may provide a window to access this hard to reach population, which would increase intervention uptake. Findings from a recent qualitative study in Canada support this idea [58]. The authors found that both physicians and parents engaged with and welcomed the idea of childhood obesity prevention interventions based within the primary care setting. However, two systematic reviews from the US that assessed paediatric primary care-based obesity interventions found that the majority of studies were based on obesity treatment, rather than focusing on obesity prevention [59, 60]. This signifies that the evidence base regarding childhood obesity prevention in primary care is insufficient in terms of study quantity and quality, which highlights a need for a greater number of randomised control trials based in primary care that assess obesity prevention interventions.

### Strengths and limitations

This is the first qualitative study specifically designed to explore potential explanations for the link between informal childcare and childhood obesity in children aged 0–5 years as evidenced by previous epidemiological studies in various countries. The study also generated rich insights into potential components and delivery strategies of future interventions. These findings could inform the development of tailored obesity prevention strategies targeted to informal caregivers and parents of children aged 0–5 years in this country. The suggestion for delivering interventions (e.g. educational information) to this difficult to reach target audience through existing points of contact with health care providers may be useful for example for, general practitioners, health visitors, and nurses who have direct and regular contacts with children under the age of five and their families. Moreover, multiple steps were taken to ensure the credibility of the results. These included involving a number of different researchers with mixed disciplinary backgrounds and experience in the data analysis process, and reporting the analysis methods and results transparently. Finally, gaining opinions from two sources, parents and carers, would allow tailored development of future interventions to both groups’ needs and wants, this may help uptake and success of the intervention in the future.

However, the results of this study should be interpreted with certain limitations. Firstly, despite numerous efforts to recruit both parents and carers into this study, only 14 participants took part in this study within our project’s limited timeframe. However, all the focus groups generated rich data and as discussed earlier, our findings are largely consistent with relevant, previously published literature. This indicates a level of validity of the study. Challenging recruitment has also provided valuable lessons for future studies whose success depend on the participation of informal carers. Effective recruitment strategies included advertising via social media, University mailing lists and word of mouth. Secondly, participants were mainly of Caucasian origin, thus potential cultural variations in the results could not be explored fully. This is significant due to the wide cultural diversity of the British population. Thirdly, all participants reported to have university or college level education so they might be more comfortable with educational interventions, compared to those with a lower level of educational achievement. Future studies should aim to include participants from more varied ethnic and socio-economic backgrounds.

Further research should explore the views of primary and community health care providers (e.g. antenatal midwives, health visitors, GPs and nurses) regarding potential opportunities and barriers for them to support or deliver an intervention programme that targets children under the age of 5 years in informal care for obesity prevention in those children.

## Conclusions

This qualitative study, with both informal carers and parents of children aged 0–5 years, provided novel insights into the informal care arrangement. Potential explanations for the previously evidenced link between informal care and childhood obesity were identified. Conflicting opinions between older members of the family and healthcare professionals made adoption of healthy feeding practices, such as breastfeeding and baby-led weaning, difficult and almost impossible for some parents if they were not getting the support they needed. Parents reported a balancing act between receiving childcare for their children but in return losing control over the child’s feeding and activity levels, as they felt indebted to their carer. Both parents and carers identified that children in informal care have less physical activity than their peers in formal care, potentially due to advancing age of many informal carers, and eat more energy-dense snacks. Our findings highlight that education targeted towards informal carers will help them to support the parents, and also to prevent obesity in the children in their care. We propose that future childhood obesity prevention interventions aimed at this population are delivered via existing primary care platforms (such as midwife, health visitor or GP appointments) in order to provide a cost-effective approach to reach as many families as possible. Future research should explore the feasibility and acceptability of this intervention idea to healthcare professionals who have contact with children under five and their families.
